# Chemopreventive Effect of an In Vitro Digested and Fermented Plant Sterol-Enriched Wholemeal Rye Bread in Colon Cancer Cells

**DOI:** 10.3390/foods13010112

**Published:** 2023-12-28

**Authors:** Diego Miedes, Antonio Cilla, Amparo Alegría

**Affiliations:** Nutrition and Food Science Area, Faculty of Pharmacy, University of Valencia, Av. Vicente Andrés Estellés s/n, 46100 Burjassot, Spain; diego.miedes@uv.es (D.M.); amparo.alegria@uv.es (A.A.)

**Keywords:** plant sterols, antiproliferative, colonic fermentation, short chain fatty acids, Caco-2 cells, rye

## Abstract

Diet is crucial for the prevention of colorectal cancer. Whole grains are the source of beneficial compounds for this, such as fiber. The enrichment of wholemeal rye bread with plant sterols (PSs) could increase its beneficial effects. This study aimed to assess the potential antiproliferative effect of this enriched food on colon adenocarcinoma cells (Caco-2) compared with a non-enriched one. After a human oral chewing, simulated semi-dynamic gastrointestinal digestion and colonic fermentation in a simgi^®^ system, fermentation liquids (FLs) obtained were used as treatment for cells. Cytotoxicity assay showed that samples diluted 1/5 (*v*/*v*) with DMEM are not toxic for non-tumoral cells, whereas they damage tumoral cells. Samples with PS (FLPS) produced a higher chemopreventive effect (vs. blank) in MTT and apoptosis assays, as well as higher gene expression of *TP53* and *Casp8*. Nevertheless, FL0 (without PS) produced a higher chemopreventive effect in a cell cycle and reduced glutathione and calcium assays, besides producing higher gene expression of *Casp3* and lower *CCND1.* The distinct antiproliferative effect of both FLs is attributed to differences in PSs, short chain fatty acids (lower concentration in FLPS vs. FL0) and antioxidant compounds. These results may support wholemeal rye bread consumption as a way of reducing the risk of colorectal cancer development, although further research would be needed.

## 1. Introduction

In 2020, colorectal cancer (CRC) produced almost 1 million deaths per year worldwide, representing the second cause of cancer death [1]. One of the most important risk factors for this disease is diet, which can interfere with colonic microbiota, and increase or reduce the risk of tumor formation. The intake of fruits and vegetables, rich in bioactive compounds (fiber, plant sterols (PSs), phenolic compounds, flavonoids, and sulfur compounds), is related with a lower risk of CRC development [2,3].

In many cases, prior to exerting their health beneficial effects, bioactive compounds orally ingested are released from the food matrix and/or transformed during digestion through the gastrointestinal tract. In this sense, simulated gastrointestinal digestion methods are widely used in order to evaluate the bioaccessibility and bioactivity of complex food matrices containing bioactive compounds since they can mimic human digestion conditions. The dynamic ones allow us to reproduce the human digestive conditions (pH gradient, peristalsis, or enzyme secretion) more accurately than static methods. Among the dynamic gastrointestinal digestion methods, the simgi^®^ model consists of a multi-compartmental and continuous system, that includes five reactors (for the stomach, small intestine, and ascending, transverse, and descending colon) [4].

Dietary fiber is known for its protective effect, with many epidemiological studies supporting its preventive effect on CRC [5,6,7]. Fiber intakes superior to 20 g/day are related to a lower risk of CRC. The metabolization of fiber by colonic microbiota produces short chain fatty acids (SCFAs), which are responsible for a lower tumor risk. Furthermore, fiber prevents constipation, a major risk factor in the development of CRC. It also blocks the formation of toxic bile acids such as deoxycholic acid [2]. One of the most important fiber sources are wholemeal cereals. White wheat bread is the most common bread in developed countries, so whole grain alternatives like wholemeal rye bread are of interest. This type of bread contains high amounts of fiber (20.6 g fiber/100 g of whole grain flour [8]), mainly arabinoxylans.

Plant sterols (PSs) have also shown a protective effect against cell proliferation in the colonic tissue. The chemopreventive effect is mediated by apoptosis induction and cell cycle regulation of tumoral cells, via NF-κB inhibition, or a decrease in gene expression of Bcl-2 and cyclin D1, among others. This action is attributed to their low intestinal absorption [9,10]. In this sense, previous studies have assessed the chemopreventive effect of estimated colonic concentrations of various PSs [11,12], and of PS-enriched beverages [13].

The chemopreventive effect of fermentation liquids (FLs) obtained after simulated gastrointestinal digestion and fermentation has been evaluated treating colon adenoma cells (LT97) with several fiber-rich food matrices, such as nuts [14,15,16] and cereals [17,18,19,20,21]. The presence of fiber and the subsequent SCFA formation was the main contributor to the abovementioned chemopreventive effect of those FLs. However, these effects of FLs from foods enriched with PSs have not been evaluated.

The main objective of this study is to evaluate the antiproliferative effect at the colonic level of FL from PS-enriched wholemeal rye bread (PSRB), compared to FL from non-enriched wholemeal rye bread (NERB). To unravel the potential differences between the digested and colonic fermented samples, the focus of the study settled on the influence of PSs, the products derived from the colonic fermentation of the bread (SCFA and PS metabolites) and their total antioxidant capacity (TAC).

## 2. Materials and Methods

### 2.1. Materials

A measure of 2,2′-Azobis(2-methylpropionamidine) dihydrochloride (AAPH), bovine serum albumin, 2′,7′-dichlorofluorescein diacetate (DCFDA), dimethylsulfoxide (DMSO), 3, (4,5-dimethylthiazol-2-yl)-2,5-diphenyl-tetrazolium bromide (MTT), Fluo 3-AM, fluorescein sodium salt, 5-fluorouracile (5-FU), Folin–Ciocalteu reagent, gallic acid, monoclonal anti-ceramide antibody produced in a mouse (primary antibody), goat anti-mouse IgM + IgG + IgA anti-body, F(ab′)2, FITC conjugate (secondary antibody), propidium iodide (PI), ribonuclease A (RNase A), sodium carbonate (Na_2_CO_3_), and Trolox were purchased from Merck LifeScience S.L.U. (Madrid, Spain). A measure of 5-Chloromethylfluorescein diacetate (Green CMFDA) was acquired from Abcam (Cambridge, UK). FITC Annexin V Apoptosis Detection Kit I was purchased from BD Biosciencies (San Jose, CA, USA). TRIzol^TM^ reagent was purchased from Invitrogen™ (Carlsbad, CA, USA). A ReliaPrep™ RNA miniprep system kit was acquired from Promega (Madison, WI, USA). A TaqMan™ reverse transcription kit and PowerUp™ SYBR™ green master mix were purchased from Applied Biosystems™ (Foster city, CA, USA). D-MEM + GlutaMAX^TM^ (4.5 g/L glucose), MEM non-essential amino acids (MEM NEAA) solution (100×), HEPES buffer solution (1 M), antibiotic solution (10,000 U/mL penicillin and 10,000 µg/mL streptomycin), antimycotic solution (250 µg/mL amphotericin B), fetal bovine serum (FBS), PBS pH 7.4 (1×) and trypsin-EDTA solution (2.5 g/L trypsin and 0.2 g/L EDTA) were acquired from Gibco™ (Scotland, UK). Absolute ethanol was obtained from Panreac (Barcelona, Spain). Measures of 2-Propanol and chloroform were supplied by Scharlau (Barcelona, Spain). Deionized water was obtained using a Milli-Q water purification system (Millipore™, Bedford, MA, USA).

### 2.2. Samples

Both a PSRB (containing 1.3 g of PS/portion of 80 g) and a NERB were manufactured. The manufacturing process and descriptions of the PS source ingredient were previously described [22]. Table 1 summarizes the nutritional information of both breads.

### 2.3. Semi-Dynamic In Vitro Digestion and Colonic Fermentation

The dynamic gastrointestinal digestion and colonic fermentation system used was simgi^®^, developed by CIAL (CSIC-UAM) (Madrid, Spain), described by Tamargo et al. [23] with some modifications. This system consists of five compartments (stomach (S), small intestine (SI), ascending colon (AC), transverse colon (TC), and descending colon (DC)) with a maintained temperature (37 °C). Furthermore, it has a constant flow of enzyme solutions, as well as NaOH and HCl to maintain the pH at each stage of digestion (S: 1.8; SI: 7; AC: 5.6; TC: 6.3; DC: 6.8).

Gastrointestinal digestion was carried out in a reactor without peristaltic movements and without emptying into the small intestinal compartment due to the high quantity of fiber of the rye bread in the oral bolus. However, the progressive addition of enzymes and solutions to maintain pH was performed in the same way. In the remaining compartments (colonic vessels), pipes connect them, and the peristaltic valve pumps were automatically controlled.

The fecal sample for the colonic fermentation was obtained from a healthy donor with the following exclusion criteria: the donor had not received any anabolic, hormonal, antibiotic or hypocholesterolemic treatment; consumed herbal products, enriched foods, vitamin, carotenoid, pro/prebiotics, PS, or phytoestrogen supplements; suffered any acute inflammation or gastrointestinal diseases; taken any chronic medication; and was not vegan or vegetarian.

**Figure 1:**
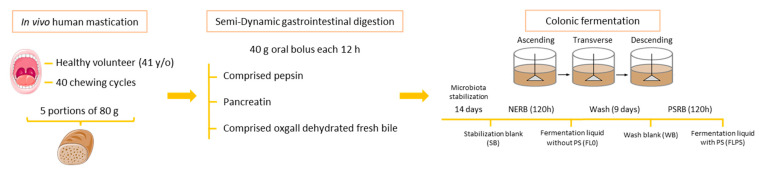
Schematic diagram of the semi-dynamic gastrointestinal digestion and colonic fermentation. NERB: non-enriched wholemeal rye bread; PSRB: plant sterol-enriched wholemeal rye bread.

Figure 1 shows a schematic diagram of the gastrointestinal digestion and colonic fermentation process and sample collection. Samples of the FL from the DC vessel corresponding to time 0 (stabilization blank, SB) were taken at the end of the microbiota stabilization period of 13 days, before digesting any bread.

Prior to the dynamic digestion with simgi^®^, a human oral phase was performed. Five individual breads, representing portions of PSRB and NERB (81.45 ± 1.14 g) were chewed as described previously by Faubel et al. [24] and five independent oral boluses were obtained for each type of bread. Due to the difficulties presented by the gastrointestinal digestion of the oral boluses in the simgi^®^, a semi-dynamic gastrointestinal digestion of these oral boluses corresponding to one intake (40 g PSRB or NERB) was performed. To feed the simgi^®^ during 120 h (5 days), gastrointestinal digesta were added twice each day, representing a consumption of one portion of bread per day (80 g of NERB or PSRB). After the microbiota stabilization period, the AC was fed with the gastrointestinal digesta corresponding to the NERB (145 mL digesta from 40 g bread) (representing the first consumption of bread for one day) and 8 h later, another amount of intestinal digesta (145 mL) (corresponding to the next 40 g of daily consumption of bread) was added. Aliquots of the fermentation liquid without plant sterols (FL0) were taken at 120 h at the end of the DC and stored at −20 °C.

Next, a washout period was performed during 9 days in order to stabilize the microbiota and remove the sterol residue from the NERB, and aliquots of the wash blank (WB) were taken at the end of this period. Then, the AC was fed with the PSRB in the same way as for the NERB. Aliquots of the fermentation liquid with plant sterols (FLPS) were collected from the DC at 120 h and stored at −20 °C.

### 2.4. Cell Culture

CCD-18Co (normal colon fibroblasts) and Caco-2 (colorectal adenocarcinoma) cells were acquired from the American Type Culture Collection (CRL-1459 and HTB-37, respectively) (Rockville, MD, USA). Both cell lines were cultured in 75 cm^2^ flasks (Corning™ Falcon™) with D-MEM supplemented with 10% (*v*/*v*) FBS, 1% (*v*/*v*) HEPES, 1% (*v*/*v*) MEM NEAA, 1% (*v*/*v*) antibiotic solution, and 1% (*v*/*v*) antimycotic solution. Cells were maintained at 37 °C in a humidified atmosphere (95% relative humidity, RH) with 5% (*v*/*v*) of CO_2_.

The four samples (SB, FL0, WB and FLPS) were centrifuged (3100× *g*/15 min/4 °C) and the supernatants were stored. To avoid any cytotoxicity artifact, these supernatants were diluted with D-MEM (1/5, *v*/*v*), filtered through 0.45 µM (PTFE) and stored at −20 °C.

For experimental studies, Caco-2 (passages 19–38) and CCD-18Co (passages 8–15) cells were dissociated from the flask with trypsin-EDTA. Subsequently, cells were plated at 5 × 10^4^ cells/cm^2^ onto 96-well (MTT assay), 24-well (cytofluorometric analysis) or 6-well plates (qPCR assays) (Costar Corp., Cambridge, MA, USA) and incubated (37 °C, 95% RH, 5% (*v*/*v*) CO_2_) during 24 h with a culture medium. After that, the cells were treated with the samples for 24 h because this reflects a realistic estimate of human intestinal exposure to dietary compounds. However, gene expression assays (qPCR) were performed at 3 and 6 h since transcriptional modifications are initiated during the early exposure to treatment. Untreated cells were used as a control, and 5-FU (25 μM) was used as a positive control, owing to its cytotoxic effect on colon cancer cells.

### 2.5. MTT Cytotoxicity Assay

The MTT is based on the formation of purple formazan crystal (in active cells) from yellow tetrazolium salt (MTT), allowing us to determine the cell metabolic activity [25]. Cells were incubated with a MTT solution (4 h, 37 °C, 95% RH, 5% (*v*/*v*) CO_2_) and the formazan crystals were solubilized with DMSO. Absorbance was measured with a multi-well spectrophotometer (Synergy H1 microplate reader, BioTek, Agilent, Santa Clara, CA, USA) (570 nm, background subtraction 690 nm), and was correlated with cell viability. Results were expressed as % of viability compared to cell control.

### 2.6. Detection of Apoptosis: Annexin V/IP Assay

The cellular state was evaluated with the two-dimensional gating method of an FITC-Annexin V kit, which detects the externalization of phosphatidylserine; this represents an apoptotic event [26]. It allows us to differentiate between viable (annexin and PI negative), early apoptotic (annexin positive and PI negative), late apoptotic (annexin and PI positive) and necrotic (annexin negative and PI positive) cells and was measured by flow cytometric analysis (FACS Verse, BD Biosciences). At least 1 × 10^4^ events were analyzed for each sample.

### 2.7. Cell Cycle Progression

The proportion of the different stages of the cell cycle was evaluated using PI, which binds to the DNA and allows us to differentiate between cells with a normal DNA amount (G0/G1 phase) and cells with double the DNA amount (G2/M phase) [26]. The S phase was placed in the intermediate of these and the apoptotic cells, whose DNA content is lower, were placed as the subG1 phase. Cells were incubated with ethanol:PBS 70:30 (*v*/*v*) and after that with a mix of RNase A and PI (40 and 100 µg/mL, respectively in PBS) (30 min, 37 °C, 95% RH, 5% (*v*/*v*) CO_2_). The fluorescence was evaluated with flow cytometry (FACS Verse, BD Biosciences). At least 1 × 10^4^ events were analyzed for each sample.

### 2.8. Determination of Ceramide Levels

The levels of ceramide were evaluated using a goat anti-mouse, polyclonal, FITC, secondary antibody after incubation (60 min/37 °C/5% CO_2_/95% relative humidity) with a mouse monoclonal anti-ceramide antibody [27]. After centrifugation (450 Santa Clara *g*/5 min/4 °C), and a PBS wash, cells were incubated with 100 µL of secondary antibody (30 min/37 °C/5% CO_2_/95% relative humidity), which binds to intracellular ceramide and can be analyzed by flow cytometry (FACS Verse, BD Biosciences). At least 1 × 10^4^ events were analyzed for each sample.

### 2.9. Levels of Reactive Oxygen Species (ROS)

The measurement of ROS was performed with dichlorofluorescein diacetate (DCFDA), which is oxidized by ROS, producing fluorescence. Briefly, cells were suspended with DCFDA (1 μM, final concentration) and incubated in darkness (30 min/37 °C/5% CO_2_/95% relative humidity). After centrifugation (450 × *g*/5 min/25 °C), the pellet was suspended in 300 μL of PBS and analyzed by flow cytometry (FACS Verse, BD Biosciences) [11]. At least 1 × 10^4^ events were analyzed for each sample.

### 2.10. Intracellular Reduced Glutathione (GSH)

The content of GSH was determined with CMFDA, a specific dye able to bind to nonprotein thiol (as GSH). Briefly, the CMFDA was mixed with 500 μL of cell suspension in a cytometer tube (1 μM, final concentration). Cells were incubated with the dye (40 min/37 °C/5% CO_2_/95% relative humidity), centrifuged (450× *g*/5 min/25 °C), suspended in 300 μL of PBS, and analyzed by flow cytometry (FACS Verse, BD Biosciences). At least 1 × 10^4^ events were analyzed for each sample.

### 2.11. Intracellular Calcium Levels

Levels of intracellular calcium were determined using Fluo-3AM probe [28]. Fluo-3AM can bind to calcium and emit fluorescence proportionally to the calcium content. Briefly, cells were harvested with trypsin (2.5 g/L), centrifuged (450× *g*/5 min/25 °C), suspended in 500 μL a Fluo-3AM in PBS solution (2 μM) and incubated for 40 min (37 °C/5% CO_2_/95% relative humidity). The supernatant was removed after centrifugation (450× *g*/5 min/25 °C), 400 μL of PBS was added to each cytometer tube, and analyzed by flow cytometry (FACS Verse, BD Biosciencies). At least 1 × 10^4^ events were analyzed for each sample.

### 2.12. Evaluation of Gene Transcription by qPCR

To evaluate the expression of the regulatory genes of the apoptosis and the cell cycle, a relative quantification of its messenger RNA (mRNA) levels was performed using qPCR prior retrotranscription (RT) to complementary DNA (cDNA).

#### 2.12.1. RNA Extraction

The extraction was performed by isolating and purifying the RNA with the TRIzol^TM^ reagent. After the culture medium was removed, 500 µL of TRIzol^TM^ reagent was added and cells were harvested with cells scrapers (Sarstedt^®^, Nümbrecht, Germany), and after 5 min, they were harvested again. The cell lysate was transferred to a 1.5 mL Eppendorf^®^ tube, and the whole process was repeated. A total of 200 µL of chloroform was added and the tubes were centrifuged for 15 min at 4 °C and 12,000× *g* (Sigma^®^ 3K15). Then, the supernatants were transferred to a new tube and 170 µL of isopropanol was added. The RNA was purified using a ReliaPrep™ RNA Miniprep System kit according to the technical manual provided by the manufacturers (but 2 min centrifugation). The concentration and purity of the RNA was determined with a micro-volume spectrophotometer (NanoDrop Lite, Thermo Fisher Scientific™, Waltham, MA, USA). The extract was maintained at −80 °C until analysis.

#### 2.12.2. Primer Design

Primers for each gene were designed using Primer-BLAST software of NCBI. The default criteria were maintained for a PCR melting temperature (Tm) (min 57, opt 58, max 60, and max Tm difference 0.5 °C) and product size (150 to 200 base pairs). The sequence of the target and reference gene primers as well as the amplification efficiency are shown in Table 2.

#### 2.12.3. Retrotranscription

The RT was carried out using a TaqMan™ Reverse Transcription kit, according to the protocol provided by the manufacturers (25 °C for 10 min, 48 °C for 30 min, 95 °C for 5 min and stabilization at 16 °C), obtaining a final concentration of 30 ng/μL of cDNA.

#### 2.12.4. Quantitative Real-Time PCR

Amplification reactions were performed with a StepOne Plus Real-time PCR instrument. SYBR™ Green detection chemistry was used for the reaction, carried out in a MicroAmp™ Fast Optical 96-Well Reaction Plate 0.1 mL (Applied Biosystem™). Each reaction (10 μL, three technical replicates) was composed of 45 ng cDNA, 5 μL of PowerUp™ SYBR™ Green Master Mix, and 2 μL of solution of primer pair (900 nM of both primers). There was an initial holding stage at 95 °C for 5 min, followed by 50 cycles of denaturation at 95 °C for 10 s, annealing at 60 °C for 30 s and an elongation at 72 °C for 15 s. After amplification, melting curve analysis was performed over a gradient (+0.3 °C/min) extending from annealing to the denaturation temperature.

#### 2.12.5. Gene Expression Analysis

Changes in gene expression in apoptosis-related (*BAX*, *BCL2*, *CASP3*, *CASP8*, and *CASP9*) and cell cycle-related genes (*CDKN1A*, *TP53*, *CCND1*, and *CCNE1*) were assessed using the 2^−ΔΔCq^ method [29]. The ACTB gene was used as a reference gene since its expression remains constant across the different conditions.

### 2.13. Total Antioxidant Capacity

Since no standardized TAC method exists, at least two different methods with different mechanisms of action, hydrogen atom transfer (HAT) reaction mechanism (ORAC) and electron transfer (ET) based assay (Folin–Ciocalteu method) were performed [30].

#### 2.13.1. Oxygen Radical Absorbance Capacity (ORAC) Method

The ORAC method was performed following Ou et al. [31], with modifications. A mixture of 80 μL of fluorescein (1 μM), 80 μL of the samples (1/5 DMEM diluted supernatants of SB, FL0, WB, or FLPS, or Trolox standard) and 40 μL of an AAPH solution in PBS (250 mM) was added. The fluorescence intensity was measured every min during 90 min (λexc = 485 nm and λem = 520 nm) using a multi-well spectrophotometer (Victor^3^ multi-label counter, Perkin-Elmer, Waltham, MA, USA) and the area under the curve was calculated. The results were compared with a Trolox standard (20 μM) and were expressed as µM Trolox.

#### 2.13.2. Total Phenolic Content (TPC)

The TPC was determined following the Folin–Ciocalteu method [32]. A mixture of 3 mL of a Na_2_CO_3_ solution (2%, *v*/*v*), 100 μL of sample (1/5 DMEM diluted supernatants of SB, FL0, WB, or FLPS) and 100 μL of Folin–Ciocalteu reagent was incubated for 1 h. Then, the absorbance of samples and a gallic acid calibration curve was measured with a spectrophotometer at 765 nm (Lambda 365 uv/vis spectrophotometer, Perkin-Elmer, Waltham, MA, USA). Results were expressed as mg gallic acid equivalents (GAE)/L.

### 2.14. Short Chain Fatty Acids Determination

Acetic, propionic, butyric and valeric acid from all samples were analyzed (n = 2) by GC-FID (Agilent 6890A, Agilent), using helium as the carrier gas (1.5 mL min^−1^) and a column DB-WAXtr (60 m, 0.325 mm × 0.25 µm). The temperature started at 50 °C for 2 min, at 15 °C min^−1^ was raised until 150 °C, at 5 °C min^−1^ was raised until 200 °C, at 15 °C min^−1^ was raised until 240 °C, and kept for 20 min. Results were expressed as a concentration (mM) of SCFA.

### 2.15. Statistic Analysis

All assays were carried out in two independent experiments (at least n = 3 per assay). Shapiro−Wilk was used to check normality and a Levene test to check homoscedasticity. One-way analysis of variance (ANOVA), followed by a Tukey’s post hoc test, was applied to determine statistically significant differences between treatments (*p* < 0.05). The program used for statistical analysis was Graphpad Prism 8.0.2 (GraphPad Software Inc., San Diego, CA, USA).

## 3. Results

### 3.1. Cytotoxicity

The selectivity of the cytotoxic effect of the samples on tumoral cells was assessed (see Table 3). No cytotoxic effect was observed on the non-tumoral cell line after all treatments with the three dilutions assayed for 24 h. It is possible that the content of the SCFAs may be the main contributor. SCFAs (butyrate mainly) are a relevant source of energy for colonocytes, and a higher content of SCFAs could improve normal cell development. On the other hand, in the tumoral cell line only 1/5 dilution did increase the cytotoxicity significantly (*p* < 0.05). The viability reduction after treatment with FL compared to their blanks was higher with FLPS (34%) compared to FL0 (15%).

Considering these results, it was decided to use the 1/5 dilution of the treatments for the subsequent assays only with tumoral cells. In addition, the non-tumoral cell line was no longer used, as its only purpose was to ensure that the effect of treatments on tumoral cells does not impact on the normal colonic epithelium.

### 3.2. Apoptosis

**Figure 2:**
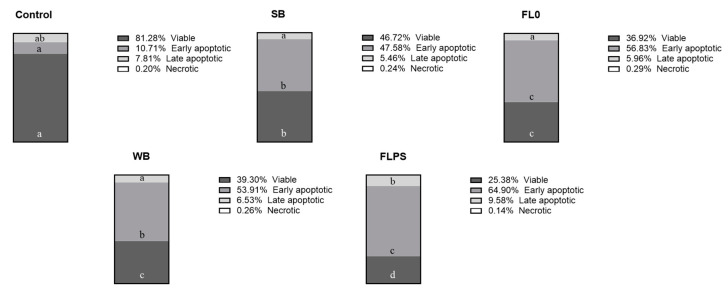
Distribution of Caco-2 cells in the different cell states after 24 h of treatment with samples (1/5 dilution). Data are shown as mean (n = 3). Different letters (a–d) in the same cell state (viable, early or late apoptotic, and necrotic) indicate statistically significant differences between treatments (columns) (*p* < 0.05). SB: stabilization blank; FL0: fermentation liquid without plant sterols; WB: wash blank; FLPS: fermentation liquid with plant sterols.

The cell status distribution after treatment with the samples is represented in Figure 2. The % of apoptotic cells (the sum of early and late apoptotic cells) was higher in both blanks (53 and 60% in SB and WB, respectively) compared to the control (19%). This is increased after treatment with FL samples (63 and 74% in FL0 and FLPS, respectively). Comparing FLs with their respective blanks, viable cells were reduced to a greater extent with FLPS (by 35%) compared to FL0 (by 21%). Early apoptotic cells were increased similarly (by 19%). Otherwise, late apoptotic cells were further increased with FLPS (by 47%) compared to FL0 (by 9%).

### 3.3. Cell Cycle

**Figure 3:**
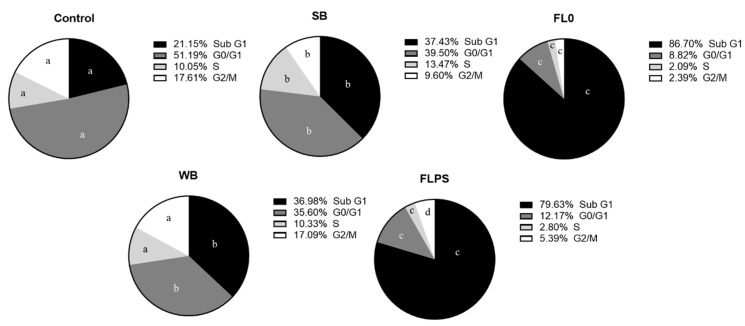
Distribution of Caco-2 cells in the different phases of the cell cycle after 24 h of treatment with samples (1/5 dilution). Data are shown as mean (n = 3). Different letters (a–c) in the same phase (Sub G1, G0/G1, S, and G2/M) indicate statistically significant differences between treatments (circles) (*p* < 0.05). SB: stabilization blank; FL0: fermentation liquid without plant sterols; WB: wash blank; FLPS: fermentation liquid with plant sterols.

The cell distribution in the different phases of the cell cycle is represented in Figure 3. All treatments produced a statistically significant increase (*p* < 0.05) (vs. control) of cells in Sub G1 phase (marker of apoptosis). Furthermore, an increase in the Sub G1 population after treatment with FL (vs. respective blanks) (by 132 and 116% with FL0 and FLPS, respectively) was observed. Consequently, the proportion of all subsequent phases was significantly reduced, and none showed an arrest.

These data confirm the results obtained in the Annexin V/IP assay. This means that FL could act mainly through an induction of apoptosis and a reduction in cell proliferation.

### 3.4. Cell Death Mechanisms

**Figure 4:**
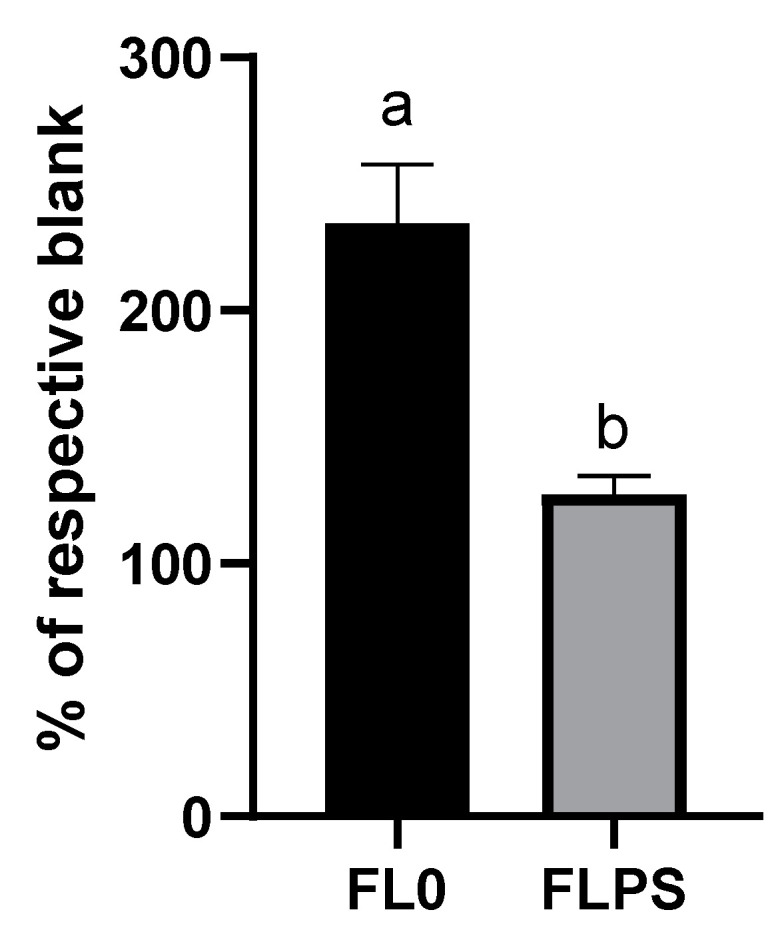
Levels of intracellular calcium in Caco-2 after 24 h of treatment with samples (1/5 dilution). Data are shown as mean ± standard deviation (n = 3). Different letters (a, b) show statistically significant differences between FL0 and FLPS (*p* < 0.05). FL0: fermentation liquid without plant sterols; FLPS: fermentation liquid with plant sterols. Respective blanks are stabilization blank for FL0 and wash blank for FLPS.

The next step was to delve into the mechanisms by which cell death occurs. The cellular levels of ceramide (the second messenger in activating the apoptosis cascade) were not increased after treatment with FL (vs. respective blanks) (Supplementary Figure S1). Both blanks did produce an increase in the levels, compared to the control. This fact could indicate a possible cytotoxic effect derived from gastrointestinal digestion and colonic fermentation reagents, mediated by ceramide. Nevertheless, intracellular calcium levels did show an increase (*p* < 0.05) after FL treatment compared to their respective blanks (Figure 4). The increase observed was higher with the FL0 treatment (134%), while with the FLPS it was more moderate (27%).

### 3.5. Cell Redox Status

**Figure 5:**
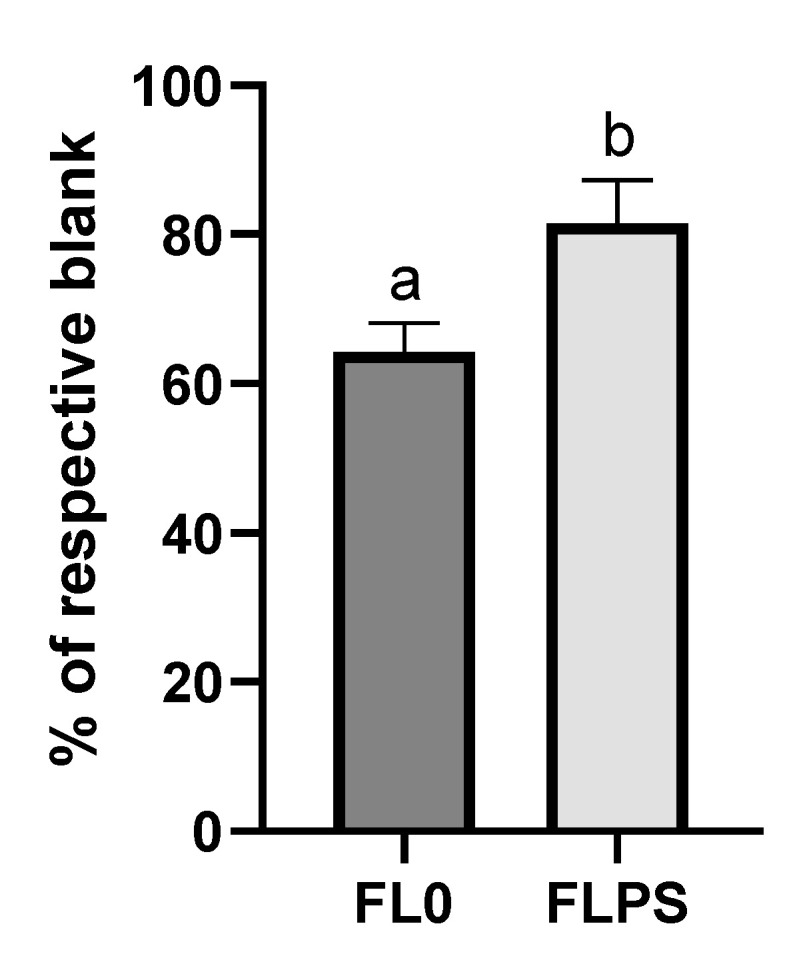
Levels of intracellular glutathione in Caco-2 after 24 h of treatment with samples (1/5 dilution). Data are shown as mean ± standard deviation (n = 3). Different letters (a, b) show statistically significant differences between FL0 and FLPS (*p* < 0.05). FL0: fermentation liquid without plant sterols; FLPS: fermentation liquid with plant sterols. Respective blanks are stabilization blank for FL0 and wash blank for FLPS.

The alteration in the cell redox status can promote apoptosis in tumoral cells. In this sense, intracellular GSH levels showed a statistically significant reduction (*p* < 0.05) after treatment with both FLs (vs. blanks). The reduction was higher with the FL0 treatment (by 36%) compared to with the FLPS treatment (by 18%) (Figure 5). However, the ROS assay did not show the same trend, and no increase in ROS levels was observed after the FL treatment (Supplementary Figure S2).

### 3.6. Transcriptional Changes in Apoptosis and Cell Cycle Regulation-Related Genes

**Figure 6:**
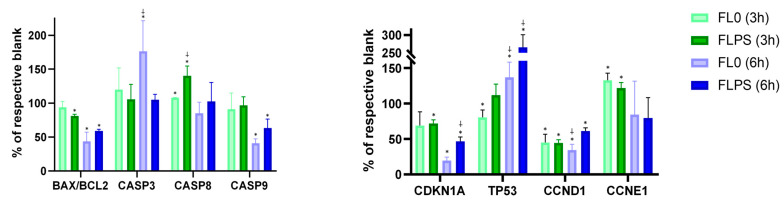
Gene expression of Caco-2 cells after 3 and 6 h of treatment with samples. Data are expressed as mean ± standard deviation (n = 9). For the same gene and same time, the asterisk (*) indicates statistically significant differences (*p* < 0.05) between the treatment and its blank. The sign ⸸ indicates statistically significant differences (*p* < 0.05) between both treatments at the same time (FL0 and FLPS). FL0: fermentation liquid without plant sterols; FLPS: fermentation liquid with plant sterols.

Figure 6 shows changes in gene expression at 3 and 6 h (vs. the respective blank). No increase was observed in the *BAX*/*BCL2* ratio, or in *CASP9* expression with the FL treatment at both durations. This fact eliminates the possibility of cell death occurring through the intrinsic (mitochondrial) pathway. However, *CASP8* expression did show a statistically significant increase (*p* < 0.05) after both FL treatments at 3 h. The increase was higher with FLPS (by 40%) compared to with FL0 (by 8%). After 6 h of treatment, there were no changes in the expression of this gene. This indicates that at 3 h, apoptosis signaling is still occurring, whereas after 6 h, cell death is already well advanced. This is confirmed with the results of the *CASP3* gene expression, which is the executor caspase being at the end of the signaling cascade responsible for carrying out apoptosis. While after 3 h of treatment, neither FL0 nor FLPS produced any increase in expression (the time when cell death signaling is still occurring), after 6 h, FL0 did increase *CASP3* expression (by 76%). In addition, despite the FLPS not producing a statistically significant increase, an upward trend can be observed, although without statistical significance. Furthermore, an increase in *TP53* expression after both FL treatments for 6 h was observed. In this case, the FLPS produced a much larger increase (by 166%) compared to the FL0 (by 37%). Furthermore, *CCND1* showed a statistically significant decrease (*p* < 0.05) with both FL and treatment durations. The reduction was higher with FL0 compared with FLPS (65 vs. 39% and 55% vs. 55% after 6 and 3 h, respectively). The inhibition of cyclin D1 means a higher regulation of the cell cycle, as this cyclin works in the early stages of the cell cycle and may imply a decrease in cell proliferation. The results are consistent with what was observed in the cell cycle assay. Conversely, *CCNE1* expression was slightly increased (at 3 h) or unchanged (at 6 h). This is consistent with the lack of cell cycle arrest previously observed. Proliferation in the present study is downregulated mainly through cell death, so cyclins that act in later phases of the cycle could not show a decrease (as is the case). Finally, *CDKN1A* showed a reduced gene expression with FL compared to blanks, although the decrease is time-dependent, showing a higher decrease after 6 h of treatment with samples compared with 3 h of treatment.

### 3.7. Total Antioxidant Capacity

After subtracting the absorbance given by DMEM in both assays (used to dilute the samples), the results were expressed as an increase in antioxidant capacity of FL vs. the respective blank. The TPC increase was statistically significant higher (*p* < 0.05) in FLPS (55.6 ± 1.7 mg GAE/L) compared to FL0 (47.8 ± 1.9 mg GAE/L). A slightly higher antioxidant capacity (*p* < 0.05) was also observed by the ORAC method in the FLPS samples. An increase (vs. blank) of 2267 ± 585 μM Trolox was observed in FLPS, whereas the increase observed in FL0 was of 2032 ± 506 μM Trolox.

### 3.8. Short Chain Fatty Acids

The concentrations of the main SCFA in the DC at 120 h after gastrointestinal digestion and colonic fermentation from all samples is summarized in Table 4. FLPS contained significantly less total SCFAs compared to FL0. Likewise, the concentration of butyrate was also lower in FLPS.

## 4. Discussion

Our results show that FLs from wholemeal rye bread (enriched or not with PS) can exert a chemopreventive effect via apoptosis, cell cycle regulation and modification of related genes. They have a cytotoxic effect on tumoral cells (Caco-2) without affecting normal cells (CCD-18co).

Diet can be a useful tool to help prevent CRC. The use of bioactive compounds such as PSs, in the context of a healthy diet may exert a chemopreventive effect [33]. The treatment of this disease is generally conducted through chemotherapeutic agents. These substances often cause various side effects such as nausea, mucositis, diarrhea, or vomiting, which can cause a worsening of the diet [34]. PSs remain highly unabsorbed at the colon [35] and perform their antiproliferative effect, targeting cell death in tumoral cells (such as the Caco-2 cell line) without damaging normal epithelial cells (such as the CCD18-Co cell line). Furthermore, the presence of fiber in the digested/fermented samples, with the subsequent formation of SCFA, together with the antioxidants, could have a synergistic action in the antiproliferative effects observed [3].

The chemopreventive effects of PSs extend to numerous types of cancer, but cancers of the digestive tract are the most relevant [5]. The low absorption of PSs, which is below 5% in healthy adults [35], produces a longer time of contact with the tissue, facilitating their effects. In this sense, stigmasterol has shown a chemopreventive effect in gastric cancer cells (SGC-7901 and MGC-803), via apoptosis induction. Stigmasterol inhibits the phosphorylation of mTOR and downregulates this pathway, which produces cell cycle arrest, activates cell apoptosis, and inhibits cell migration and invasion [36]. Furthermore, other studies have shown that ethylcoprostanol (β-sitosterol metabolite) also has a chemopreventive effect at colonic concentrations, damaging Caco-2 cells (producing 75% of early apoptosis) without negatively affecting normal cells (only 24% of early apoptosis in CCD-18Co) [37].

**Figure 7:**
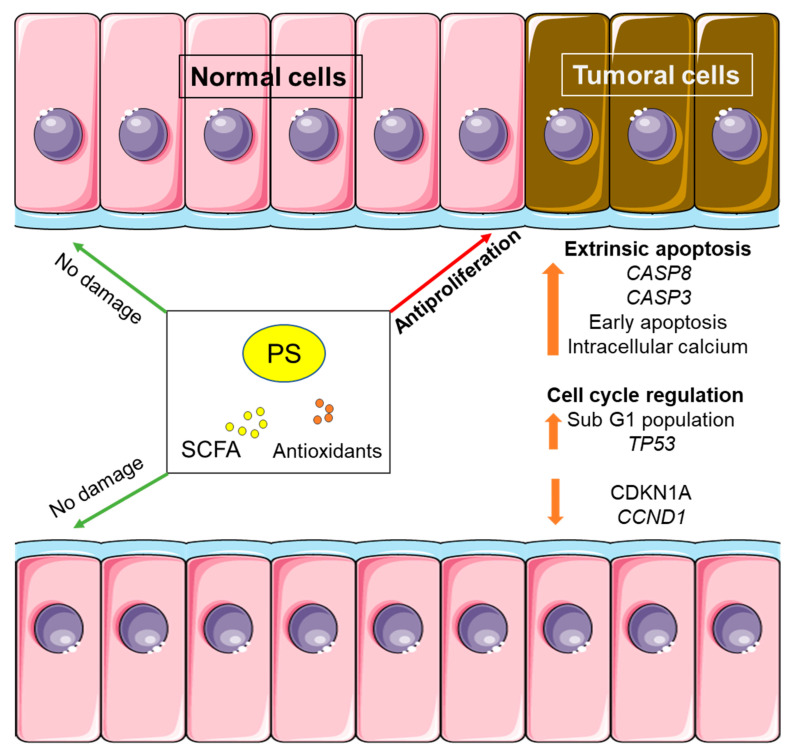
Effect of digestion and fermentation products from PS-enriched wholemeal rye bread upon normal and tumoral colon cells. PS: plant sterols; SCFA: short chain fatty acids.

Whole grain consumption is associated with less relative risk of mortality by cancer (0.85) and other non-communicable diseases (e.g., cardiovascular diseases) [38]. The main reason for this reduced risk seems to be whole grains’ high fiber content. Fiber intake is directly responsible for a lower CRC risk, as observed by the EPIC study [39]. Rye bread consumption could also be linked to a lower CRC risk compared with white wheat bread consumption, as observed in a randomized crossover trial. The protective effect can be attributed to a reduced intestinal transit, an increased fecal weight, and less total bile acid concentration in feces, highlighting a lower presence of secondary bile acids, which are potentially negative for the colonic epithelium [40]. In our study, the gastrointestinally digested and colonically fermented wholemeal rye bread (14.7 and 13.7 g fiber/100 g in NERB and PSRB, respectively) produced an antiproliferative effect at the colonic level, in line with the observational studies. The first finding was the antiproliferative effect of the products of colonic digestion and fermentation on tumoral cells. In addition, no cytotoxic effect was observed with the samples from both breads on normal colon cells. This observation confirms a selective cytotoxic effect for colonic tumoral cells by FLs (Figure 7). Therefore, the consumption of any of the wholemeal rye breads would be of interest in the primary prevention of the disease.

Two opposite trends were observed in all antiproliferative assays for certain chemopreventive parameters with an increased effect in FL0 vs. FLPS, or the contrary. These differences could be attributed to three different factors (PS, SCFA, and TAC). Firstly, the slight difference in PS content between the NERB and the PSRB. The enrichment of the bread is directly related to the higher PS content after digestion and colonic fermentation in FLPS. In this sense, in a study with Caco-2 cells, the PS at colonic concentrations (132 µM combined) exerted an antiproliferative effect after 24 h, both individually (β-sitosterol, campesterol and stigmasterol) and in combination. Furthermore, the other authors observed that their combination (as occurred in our PS ingredient and, consequently in the PSRB) had a more powerful effect, producing a decrease in cell viability of 59% compared to the control [11]. Additionally, it has been observed that the combination of PS and 5-FU enhanced the therapeutic effect that 5-FU performed on Caco-2 and HT-29 colon cancer cells. An increased apoptosis and cell cycle arrest in the S phase were responsible for this effect [12]. These findings could support the superior cytotoxic effect elicited by FLPS in MTT and Annexin V/IP assays.

Secondly, SCFA production was higher after feeding the dynamic digester with the NERB (65 vs. 18 mM in PSRB), mainly through butyrate production (29 vs. 4 mM in PSRB). A study using the simgi^®^ system showed a higher total SCFA in the control sample (gastrointestinal digestion and colonic fermentation blank) compared with food-origin samples [41]. This is in line with what we observed in our study with the WB, which contains a higher concentration of total SCFA (except butyrate) compared with FLPS. The presence of PS would modulate the microbiota (throughout the fermentation) in such a way that decreases the butyrate-producing bacteria. Also, acetate-producing bacteria may additionally decrease during fermentation due to the presence of PS, and butyrate can be generated from acetate.

The SCFA and, specifically butyrate, has demonstrated a cytoprotective effect at the colonic level [42]. Butyrate can prevent the tumor initiation by promoting the metabolization of genotoxic substances such as 4-hydroxy-2-nonenal [43]. This is possible because enzymes that participate in the biotransformation of cytotoxic compounds are induced. In addition, butyrate can promote apoptosis and slow the cell cycle progression in tumoral colonic cells (HT-29 and LT-97). Butyrate is also capable of inducing the expression of the *CASP3* gene and consequently of producing apoptosis [44]. This aligns with the results obtained in our study, where the *CASP3* gene expression of Caco-2 cells was increased with the FL0 treatment, and only a trend was observed in FLPS. The histone hyperacetylation plays a key role in the mechanism of action of butyrate. Through this pathway, it is possible to induce apoptosis in HT-29 colon cancer cells [45]. Therefore, butyrate could prevent the appearance of tumor cells and also prevent tumor progression (secondary prevention) [46]. In addition to the effect of butyrate, acetate and propionate have also shown a potential antiproliferative effect on CRC [47]. In this sense, the production of these two SCFAs from fiber by probiotic bacteria inhibited cell proliferation and promoted cell death in CRC cells (CRL-2577). Furthermore, these compounds produced cell cycle arrest, showing a clear chemopreventive effect [48]. This highlights the relevance of the probiotic activity of colonic bacteria. Their activity is crucial to produce compounds with an antiproliferative effect such as SCFA and to prevent CRC.

In vitro studies evaluating the effect of FL obtained from fiber-rich foods like cereals and nuts on tumoral cells (HT-29 and LT-97) have shown the cytotoxic effect of butyrate [17,19,20,49]. This fact reinforces the hypothesis that the production of SCFAs by the microbiota from fermentable fiber is a major cause of the chemopreventive effect of this type of food. In our study, SCFA production (and butyrate specifically) was significantly higher in FL0 than in FLPS. For this reason, FL0 may have shown a higher increase in the Sub G1 population in the cell cycle, a higher GSH reduction, and higher intracellular calcium levels.

Lastly, the TAC of the samples may be relevant to the effect they have on the colon. Given the differences observed in the antioxidant capacity assays (TPC and ORAC), it seems likely that it is relevant to the effect at the colonic level. The slightly higher antioxidant capacity of FLPSs could exert a beneficial effect on the colon tissue. It is not clear to what extent antioxidant compounds can prevent CRC, but there is evidence that has linked some antioxidants to a lower risk of CRC. As oxidative stress is a main contributor to a malfunction of the DNA mismatch repair mechanism, antioxidant compounds could reduce this malfunction, reducing the oxidative environment. Nevertheless, the influence of long-term antioxidant intake on CRC remains unclear [50].

Even though the FL produced a greater antiproliferative effect compared to the digestion and fermentation blanks, the latter also exerted cell damage compared to the control. This damage could be related to the bile acid content of both blanks. Bile acids, mainly secondary ones, have been shown to be able to inhibit cell proliferation at the colonic level. In fact, physiological concentrations of deoxycholic acid inhibit colonic cell proliferation through cell cycle arrest and apoptosis. The generation of ROS in the tumoral cells has been proposed as one of the possible factors responsible for this effect [51]. This could explain the cytotoxic effect that our digestion blanks produced on Caco-2 cells. In fact, Tamargo and colleagues observed this toxic effect of the gastrointestinal digestion and colonic fermentation blanks on HCT-116 and HT-29 colon cancer cells [41]. Since the digestion model used is the same as ours, the blanks may be similar. The damage could be related to the bile acid content of both blanks, which is related with CRC risk, as observed in a crossover trial where rye bread was able to prevent the formation of potentially negative secondary bile acids such as lithocholic acid, while beneficial ones such as ursodeoxycholic acid were increased [40]. This could explain the higher toxic effect of blanks in some assays compared with FLs.

## 5. Conclusions

With all the caution in translating in vitro pre-clinical observations to the in vivo situation, the results of the present study could suggest a potential beneficial effect of wholemeal rye bread at the gastrointestinal level due to the antiproliferative effect observed of FLs in colon cancer cells. The joint effect of PS, SCFA and antioxidant compounds could be responsible for this protective effect. The FLs from the enriched wholemeal rye bread had a higher antiproliferative effect in global end-point parameters such as cell death or MTT, probably due to the higher PS and antioxidant content. Moreover, the high fiber content from both breads could help to reduce the risk of CRC development (via SCFA production), among other potential health benefits. In this sense, the enrichment with PS could suppose a synergy since PSs themselves are able to prevent cell proliferation. This fact could make this food a suitable option for the primary and secondary prevention of CRC, as a substitute for traditional white wheat bread. Furthermore, in the context of a healthy diet, the PSRB could help to prevent additional non-communicable chronic diseases such as cardiovascular diseases due to the hypocholesterolemic effect ascribed to PSs.

## Figures and Tables

**Table 1:** Nutritional information and plant sterol content (g/100 g bread) of enriched and non-enriched wholemeal rye bread.

	PSRB	NERB
Water	32.7 ± 1.0	26.14 ± 0.02
Lipids	2.76 ± 0.02	1.16 ± 0.02
Carbohydrates	44.3 ± 0.4	51.5 ± 1.3
Soluble fiber	3.4 ± 0.1	4.0 ± 1.3
Insoluble fiber	10.3 ± 1.4	10.7 ± 0.16
Proteins	5.16 ± 0.03	5.85 ± 0.11
Ash	1.37 ± 0.04	1.47 ± 0.02
Plant sterols	1.6 ± 0.04	0.05 ± 0.001

Data are shown as mean ± standard deviation (n = 3). NERB: non-enriched wholemeal rye bread; PSRB: plant sterol-enriched wholemeal rye bread.

**Table 2:** Sequence of primers of the evaluated genes and their efficiency.

Gene	Primer Sequence (5′→3′)		Efficiency (%)
	Forward	Reverse	
*BAX*	GGGCCCACCAGCTCTGA	CCTGCTCGATCCTGGATGA	108
*BCL2*	GGATTGTGGCCTTCTTTGAG	GCCGGTTCAGGTACTCAGTC	121
*CASP3*	CCTGGTTATTATTCTTGGCGAAA	GCACAAAGCGACTGGATGAA	125
*CASP8*	CAGGCAGGGCTCAAATTTCT	TCTGCTCACTTCTTCTGAAATCTGA	121
*CASP9*	TGCTGAGCAGCGAGCTGTT	AGCCTGCCCGCTGGAT	130
*CDKN1A*	GATGAGTTGGGAGGAGGCAG	GAGCGAGGCACAAGGGTACA	140
*TP53*	CCACCATCCACTACAACTACAT	CACAAACACGCACCTCAAA	135
*CCND1*	CCCTCGGTGTCCTACTTCAA	AGGAAGCGGTCCAGGTAGTT	107
*CCNE1*	CTCCAGGAAGAGGAAGGCAA	TCGATTTTGGCCATTTCTTCA	127
*ACTB*	CTGGAACGGTGAAGGTGACA	AAGGGACTTCCTGTAACAATGCA	112

*BAX*, *BCL2*, *CASP3*, *CASP8*, *AND CASP9—*Cell apoptosis; *CDKN1A—*p21, involved in phase S of cell cycle; *TP53*—p53, cell cycle regulation; *CCND1—*cyclin D1, involved in cell cycle G1/S phase; *CCNE1—*cyclin E1, involved in cell cycle G1/S phase; *ACTB—*actin beta, housekeeping.

**Table 3:** Evaluation of cytotoxicity (MTT) of the different treatments for 24 h and with different dilutions in CCD-18Co (normal colon fibroblasts) and Caco-2 (colorectal adenocarcinoma) cells vs. control cells (both lines untreated).

	Viability (% of Control)					
		CCD-18Co			Caco-2	
Treatment	1/5	1/10	1/20	1/5	1/10	1/20
SB	97.79 ± 8.09 ^aA^	98.90 ± 7.41 ^aA^	88.56 ± 4.02 ^aA^	73.36 ± 2.49 ^aB^	82.02 ± 12.42 ^aB^	119.75 ± 17.65 ^aA^
FL0	102.70 ± 6.96 ^aA^	181.36 ± 17.18 ^bA^	175.96 ± 20.90 ^bA^	62.25 ± 2.54 ^bB^	100.33 ± 1.01^bA^	109.53 ± 7.92 ^aA^
WB	104.34 ± 6.35 ^aA^	104.22 ± 6.66 ^aA^	101.62 ± 5.78 ^aA^	98.17 ± 11.09 ^cA^	96.89 ± 7.44 ^abA^	106.35 ± 14.38 ^aA^
FLPS	105.16 ± 8.77 ^aA^	103.05 ± 11.10 ^aA^	102.32 ± 18.04 ^aA^	65.00 ± 1.72 ^bB^	97.90 ± 0.61 ^bA^	104.78 ± 20.12 ^aA^
5-FU (25µM)		96.39 ± 19.01		84.96 ± 4.91		

Data are shown as mean ± standard deviation (n = 3). Different lowercase letters (a–c) in the same column (dilution) indicate statistically significant differences between treatments for the same cell line (*p* < 0.05). Different uppercase letters (A, B) indicate statistically significant differences between cell lines with the same treatment (*p* < 0.05). SB: stabilization blank; FL0: fermentation liquid without plant sterols; WB: wash blank; FLPS: fermentation liquid with plant sterols; 5-FU: 5-fluorouracil.

**Table 4:** Concentration (mM) of short-chain fatty acids in samples in the descending colon at 120 h.

	Acetate	Propionate	Butyrate	Valerate	Total
SB	30.99 ± 0.22 ^a^	12.58 ± 0.30 ^a^	4.03 ± 0.00 ^a^	3.35 ± 0.00 ^a^	55.66 ± 0.54 ^a^
FL0	31.84 ± 1.84 ^a^	2.25 ± 0.01 ^b^	29.09 ± 0.60 ^b^	0.67 ± 0.02 ^b^	65.30 ± 1.19 ^b^
WB	40.29 ± 0.59 ^b^	15.42 ± 0.22^c^	3.79 ± 0.11 ^c^	3.54 ± 0.03 ^c^	67.34 ± 0.99 ^b^
FLPS	12.65 ± 12.65 ^c^	1.38 ± 0.01 ^d^	3.75 ± 0.05 ^c^	0.37 ± 0.00 ^d^	18.49 ± 0.06 ^c^

Data are shown as mean ± standard deviation (n = 3). Different letters in the same column (fatty acid) indicate statistically significant differences between treatments (*p* < 0.05). SB: stabilization blank; FL0: fermentation liquid without plant sterols; WB: wash blank; FLPS: fermentation liquid with plant sterols.

## Data Availability

Data presented in this study are available upon reasonable request from the corresponding author.

## Supplementary Materials

The following supporting information can be downloaded at: https://www.mdpi.com/article/10.3390/foods13010112/s1, Supplementary Figure S1: Levels of ceramide in Caco-2 after 24 h of treatment with samples (1/5 dilution). Data are shown as mean ± standard deviation (n = 3). Different letters (a, b) show statistically significant differences between FL0 and FLPS (*p* < 0.05). FL0: fermentation liquid without plant sterols; FLPS: fermentation liquid with plant sterols. Respective blanks are stabilization blank for FL0 and wash blank for FLPS. Supplementary Figure S2: Levels of reactive oxygen species (ROS) in Caco-2 after 24 h of treatment with samples (1/5 dilution). Data are shown as mean ± standard deviation (n = 3). Different letters (a, b) show statistically significant differences between FL0 and FLPS (*p* < 0.05). FL0: fermentation liquid without plant sterols; FLPS: fermentation liquid with plant sterols. Respective blanks are the stabilization blank for FL0 and the wash blank for FLPS.
