# Supplementary material for: Chemopreventive Effect of an In Vitro Digested and Fermented Plant Sterol-Enriched Wholemeal Rye Bread in Colon Cancer Cells
Source: Foods. 2023 Dec 28;13(1):112. doi: 10.3390/foods13010112 (PMC10778687; doi:10.3390/foods13010112)
Supplement: Supplementary file 1 [file foods-13-00112-s001.zip › foods-2739309-supplementary.pdf]

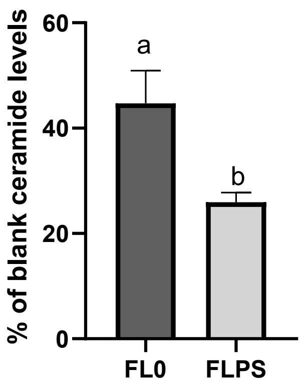

Supplementary Figure S1. Levels of ceramide in Caco-2 after 24 h of treatment with samples (1/5 dilution). Data are shown as mean ± standard deviation (n = 3). Different letters (a-b) show statistically significant differences between FL0 and FLPS ($p < 0.05$). FL0: fermentation liquid without plant sterols; FLPS: fermentation liquid with plant sterols. Respective blanks are stabilization blank for FL0 and wash blank for FLPS.

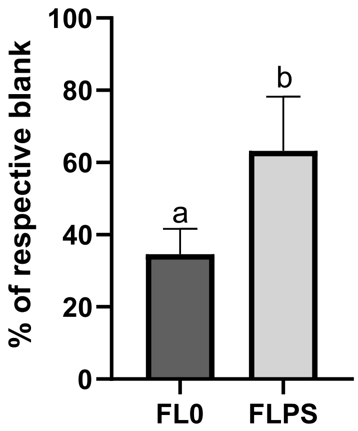

Supplementary Figure S2. Levels of reactive oxygen species (ROS) in Caco-2 after 24 h of treatment with samples (1/5 dilution). Data are shown as mean ± standard deviation (n = 3). Different letters (a-b) show statistically significant differences between FL0 and FLPS ($p < 0.05$). FL0: fermentation liquid without plant sterols; FLPS: fermentation liquid with plant sterols. Respective blanks are stabilization blank for FL0 and wash blank for FLPS.
